# Achieving 500-GHz communication over 1.2 km using an astronomical telescope with a quantum-limited superconducting receiver

**DOI:** 10.1093/nsr/nwaf222

**Published:** 2025-06-05

**Authors:** Wei Miao, Jing Li, Xianjin Deng, Jiaqiang Zhong, Yuan Ren, Daizhong Liu, Junda Jin, Binggang Ju, Yuxuan Miao, Yue He, Weijie Xu, Zhenhui Lin, Yilong Zhang, Qijun Yao, Juan Liu, Changxing Lin, Wen Zhang, Wenying Duan, Dong Liu, Kangmin Zhou, Jie Liu, Zheng Wang, Jinpeng Li, Feng Wu, Boliang Liu, Jixian Sun, Xuguo Zhang, Jibin Li, Haillong Zhang, Liang Guo, Kang Han, Zhenyu Lu, Huiqian Hao, Yuchao Dou, Zhicai Wu, Jia Quan, Yanjie Liu, Miguang Zhao, Weijie Du, Chenggang Shu, Ruiqing Mao, Shengcai Shi

**Affiliations:** Purple Mountain Observatory, Chinese Academy of Sciences, China; Purple Mountain Observatory, Chinese Academy of Sciences, China; Microsystem and Terahertz Research Center, China Academy of Engineering Physics, China; Purple Mountain Observatory, Chinese Academy of Sciences, China; Purple Mountain Observatory, Chinese Academy of Sciences, China; Purple Mountain Observatory, Chinese Academy of Sciences, China; Purple Mountain Observatory, Chinese Academy of Sciences, China; Purple Mountain Observatory, Chinese Academy of Sciences, China; Microsystem and Terahertz Research Center, China Academy of Engineering Physics, China; Microsystem and Terahertz Research Center, China Academy of Engineering Physics, China; Microsystem and Terahertz Research Center, China Academy of Engineering Physics, China; Purple Mountain Observatory, Chinese Academy of Sciences, China; Purple Mountain Observatory, Chinese Academy of Sciences, China; Purple Mountain Observatory, Chinese Academy of Sciences, China; Microsystem and Terahertz Research Center, China Academy of Engineering Physics, China; Microsystem and Terahertz Research Center, China Academy of Engineering Physics, China; Purple Mountain Observatory, Chinese Academy of Sciences, China; Purple Mountain Observatory, Chinese Academy of Sciences, China; Purple Mountain Observatory, Chinese Academy of Sciences, China; Purple Mountain Observatory, Chinese Academy of Sciences, China; Purple Mountain Observatory, Chinese Academy of Sciences, China; Purple Mountain Observatory, Chinese Academy of Sciences, China; Purple Mountain Observatory, Chinese Academy of Sciences, China; Purple Mountain Observatory, Chinese Academy of Sciences, China; Purple Mountain Observatory, Chinese Academy of Sciences, China; Purple Mountain Observatory, Chinese Academy of Sciences, China; Purple Mountain Observatory, Chinese Academy of Sciences, China; Purple Mountain Observatory, Chinese Academy of Sciences, China; Purple Mountain Observatory, Chinese Academy of Sciences, China; Changchun Institute of Optics, Fine Mechanics and Physics, Chinese Academy of Sciences, China; Changchun Institute of Optics, Fine Mechanics and Physics, Chinese Academy of Sciences, China; Changchun Institute of Optics, Fine Mechanics and Physics, Chinese Academy of Sciences, China; The 54th Research Institute of China Electronics Technology Group Corporation, China; The 54th Research Institute of China Electronics Technology Group Corporation, China; The 54th Research Institute of China Electronics Technology Group Corporation, China; Technical Institute of Physics and Chemistry, Chinese Academy of Sciences, China; Technical Institute of Physics and Chemistry, Chinese Academy of Sciences, China; Technical Institute of Physics and Chemistry, Chinese Academy of Sciences, China; School of Mathematical Sciences, Shanghai Normal University, China; School of Mathematical Sciences, Shanghai Normal University, China; Purple Mountain Observatory, Chinese Academy of Sciences, China; Purple Mountain Observatory, Chinese Academy of Sciences, China

## Abstract

500 GHz video transmission over 1.2 km is achieved using an astronomical telescope equipped with a quantum-limited superconducting SIS receiver.

High-capacity satellite/airborne-to-ground communications are essential for ensuring global connectivity and enabling high-throughput data transmission across various domains, including telecommunications, remote sensing, and space astronomy. Traditionally, these communication links have relied on microwave technologies, which are increasingly constrained by bandwidth limitations and data rate restrictions. To address these challenges, optical and terahertz (THz) communication technologies have emerged as promising alternatives [1]. While optical communication is gaining traction on satellite and airborne platforms, THz communication offers distinct advantages, such as reduced sensitivity to beam misalignment and lower susceptibility to platform vibrations. With its inherently broad bandwidth, THz communication can support data rates exceeding 100 gigabits per second (Gbps) [2–4], while maintaining minimal latency.

Recognizing the transformative potential of THz communication, significant efforts have been devoted to its advancement. However, most experiments have been confined to controlled indoor environments with short transmission distances [5], or have relied on transmitters operating at high output power levels, typically exceeding several hundred milliwatts [6,7]. As the frequency increases, generating such power becomes increasingly challenging, especially for fully-electronic transmitters, which are preferable for satellite and airborne platforms due to their compact size and low power consumption. Additionally, water vapor in the terrestrial atmosphere strongly attenuates THz signals, restricting both the usable frequency range and achievable communication distances.

To overcome these challenges, ground-based THz astronomical telescope technologies offer promising solutions for THz communication receivers. In the field of astronomy, THz telescopes like the Atacama Large Millimeter/submillimeter Array (ALMA) and the Event Horizon Telescope (EHT) have already made enormous discoveries such as the fine structure of protoplanetary disks and the recent black hole images that have had high scientific and public impact. These telescopes are situated in high-altitude regions and equipped with quantum-limited superconducting receivers that offer unparalleled sensitivity at THz frequencies, enabling broader bandwidths and longer communication distances. Notably, the exceptional sensitivity of THz astronomical receivers and telescope technologies would enable fully-electronic THz communication links on space platforms.

Here, we present the first demonstration of THz communication using an astronomical telescope as a receiver. The experiment was conducted at the Xue-Shan-Mu-Chang site on the Qinghai-Tibet Plateau, known for its unique atmospheric conditions, particularly with low precipitable water vapor (PWV) levels. Currently, the Purple Mountain Observatory of the Chinese Academy of Sciences is constructing a Xue-Shan-Mu-Chang 15-m SubMillimeter Telescope (XSMT), which will operate in the 100–500 GHz frequency range. Beyond its primary role in astronomical observations, another important goal of XSMT is to support satellite/airborne-to-ground communication links. Figure 1a presents a conceptual design for a THz communication system incorporating XSMT. Prior to its completion, a pilot portable THz telescope with a 60-cm diameter was developed to validate related technologies. This portable telescope is equipped with a newly developed 500-GHz superconductor-insulator-superconductor (SIS) receiver, which is cooled to ∼6 K with a low-power-consumption cryocooler. The optical configuration and overall system architecture are detailed in Fig. S1, while Fig. S2 presents the structure of the SIS mixer. Using this telescope, we recently carried out a point-to-point wireless communication experiment at 481.4 GHz at the site (coordinates: 37°53'54’ N and 96°40'42’ E with an altitude of 4445 m). A detailed demonstration of this achievement is provided in Supplementary Video 1. Notably, a compact fully-electronic transmitter with an emission power of only 15 µW (See Figs. S3 and S4) was utilized for this experiment.

**Figure 1.**
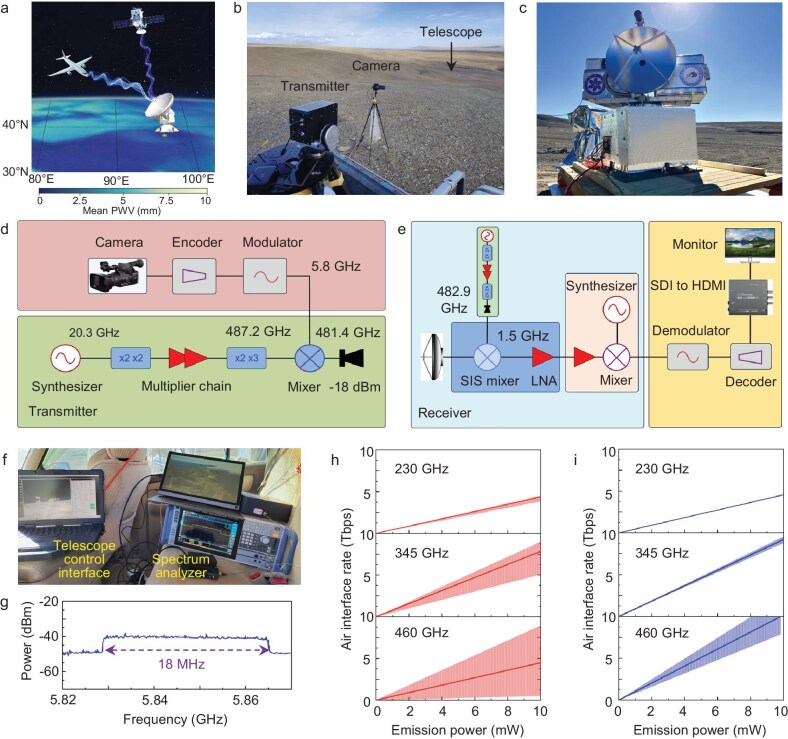
Overview and performance of a THz communication system utilizing an astronomical telescope equipped with a superconducting receiver. (a) A conceptual schematic of a future THz communication system, featuring an astronomical telescope located on the Qinghai-Tibet Plateau as a receiver for satellite/airborne-to-ground communication links. The background highlights the spatial distribution of mean PWV over the Plateau, derived from MERRA-2 reanalysis data for the winter seasons from 2013 to 2017. Blue-shaded regions indicate areas with low PWV, with darker shades corresponding to progressively lower PWV levels, emphasizing the suitability of these regions for THz communication. (b) Deployment of a 500 GHz communication system on a 4445-m highland at the Xue-Shan-Mu-Chang site. (c) Photograph of the 60-cm portable telescope, which functions as the receiver for THz communication. (d and e) Schematics of (d) the transmitter and (e) the receiver system for THz communication. In this setup, the video stream from the HD camera is encoded, modulated, and upconverted to a carrier frequency of 481.4 GHz, producing an output power of 15 μW, which is emitted through a diagonal horn. The received THz signal is down converted to 1.5 GHz by a quantum-limited SIS mixer. (f) Photograph of the communication receiving terminal. (g) Measured IF spectrum of the SIS mixer, showing a signal bandwidth of ∼18.0 MHz and a SNR of ∼10 dB. (h and i) Quartile air interface rates between the XSMT and a satellite for different emission powers across the 230, 345, and 460 GHz frequency bands under (h) annual and (i) winter-specific conditions. The solid lines represent the median data rates, while the shaded regions denote the interquartile ranges.

Figure 1b shows the deployment of the 500 GHz communication system, where the transmitter, positioned at a higher location, was separated from the 60-cm THz telescope by ∼1.2 km, with an elevation angle of ∼7 degrees. The telescope, barely visible in Fig. 1b, is shown in Fig. 1c. The atmospheric transmission at 481.4 GHz along this near-horizontal path was estimated to be 0.33, based on an atmospheric model [8] incorporating a median temperature of −2.5°C and relative humidity of 23.1% on October 1, 2024. Importantly, this transmission is nearly equivalent to that of a ground-to-space link at this site, which was simulated to 0.32 on the same date using MERRA-2 data [9].

Figure 1d and e provide schematics of the transmitter and receiver, respectively. The transmitter comprises a 20.3 GHz microwave synthesizer, a Schottky-diode-based frequency multiplier chain with a multiplication factor of 24, a high-definition (HD) camera, an encoder, a 5.8 GHz modulator, and a Schottky-diode-based THz mixer. Real-time video captured by the HD camera is compressed using H.264 encoding and modulated onto a 5.8 GHz intermediate-frequency (IF) carrier. This modulated IF signal is then upconverted to 481.4 GHz by the THz Schottky mixer. The resulting THz signal, with a power of 15 µW at 481.4 GHz, is radiated via a simple diagonal horn. The receiver is primarily composed of a 60-cm Cassegrain antenna with a gain of 65.6 dBi at 481.4 GHz and a SIS mixer. The IF signal from the SIS mixer is amplified by a cryogenically cooled low-noise amplifier with a gain of 35 dB and a noise temperature of 7 K, and a room-temperature amplifier with a gain of ∼40 dB. The amplified signal is subsequently upconverted to 5.8 GHz and demodulated into a 60 fps 1080p video stream, which is then converted by a decoder into a high-definition multimedia interface (HDMI) format for real-time display. The entire receiver system has a double sideband (DSB) noise temperature of ∼390 K (see Fig. S5). It is worth noting that for this experiment, we simply adopt off-the-shelf DJI Transmission Combo units, which integrate both encoding/modulation and decoding/demodulation functionalities, and support a maximum data rate of 40 Mbps. Figure 1f and 1g shows the receiving terminal and the received IF spectrum, respectively. The IF spectrum exhibits a signal bandwidth of ∼18.0 MHz and a signal-to-noise ratio of ∼10 dB. In this case, channel coding with forward error correction (FEC) was used to ensure stable video quality. The communication link achieved an air interface rate of ∼32 Mbps, which is constrained by the off-the-shelf modulator and demodulator. Employing higher-speed modulators and demodulators could fully exploit the large IF bandwidth (>18 GHz) of SIS mixers [10], which can significantly enhance the achievable data rate.

We also evaluated the potential of the XSMT for satellite-to-ground communications. The analysis assumes a satellite at 400 km altitude with a 1-m emission antenna and the XSMT of a system noise temperature of 200 K (with SIS operating at 4 K). According to MERRA-2 reanalysis data [9], zenith atmospheric transmission at the XSMT site has annual averages of ∼0.92, 0.74, and 0.24 at 230 GHz, 345 GHz, and 460 GHz, respectively, with winter values increasing to 0.96, 0.87, and 0.54. Figure 1h and 1i illustrate the predicted air interface rates between the XSMT and the satellite as a function of emission power. At an emission power of 10 mW, the median air interface rates under annual conditions reach 4.3 Tbps, 7.8 Tbps, and 4.5 Tbps at 230 GHz, 345 GHz, and 460 GHz, respectively. Note that these rates are derived from a theoretical link budget analysis. Achieving such high data rates, however, needs to overcome significant technical challenges such as receiver bandwidth limit, high-frequency broadband modulation schemes, and real-time high-speed signal processing on both the transmitter and receiver sides. Additionally, this evaluation was performed for zenith observations, and the air interface rate will decrease when the satellite is at a non-zenith position. The reduction in rates at 460 GHz is primarily due to decreased atmospheric transmission. In winter, these rates significantly increase due to lower water vapor, accompanied by reduced variability.

In conclusion, this work marks the first demonstration of THz wireless communication using an astronomical telescope equipped with a quantum-limited SIS mixer. Real-time HD video transmission was achieved at 481.4 GHz over a recorded distance of 1.2 km. Furthermore, this experiment is carried out with a compact fully-electronic transmitter with an emission power of only 15 µW. Theoretical analyses indicate that with the XSMT, the air interface rate on the order of Tbps can be realized. This breakthrough work marks a significant step forward for THz communication, demonstrating particularly the power of THz astronomical telescopes as ground-based receivers.

## Supplementary Material

nwaf222_Supplemental_Files
