# Supplementary material for: Achieving 500-GHz communication over 1.2 km using an astronomical telescope with a quantum-limited superconducting receiver
Source: Natl Sci Rev. 2025 Jun 5;12(8):nwaf222. doi: 10.1093/nsr/nwaf222 (PMC12239196; doi:10.1093/nsr/nwaf222)
Supplement: nwaf222_Supplemental_Files [file nwaf222_supplemental_files.zip › Supplementary Materials-final.docx]

**Supplementary information for:**

Achieving 500-GHz communication over 1.2 km using an astronomical telescope with a quantum-limited superconducting receiver

Wei Miao, Jing Li, Xianjin Deng, Jiaqiang Zhong, Yuan Ren, Daizhong Liu, Junda Jin, Binggang Ju, Yuxuan Miao, Yue He, Weijie Xu, Zhenhui Lin, Yilong Zhang, Qijun Yao, Juan Liu, Changxing Lin, Wen Zhang, Wenying Duan, Dong Liu, Kangmin Zhou, Jie Liu, Zheng Wang, Jinpeng Li, Feng Wu, Boliang Liu, Jixian Sun, Xuguo Zhang, Jibin Li, Hailong Zhang, Liang Guo, Kang Han, Zhenyu Lu, Huiqian Hao, Yuchao Dou, Zhicai Wu, Jia Quan, Yanjie Liu, Miguang Zhao, Weijie Du, Chenggang Shu, Ruiqing Mao, and Shengcai Shi

Authors to whom correspondence should be addressed: [lijing@pmo.ac.cn](mailto:lijing@pmo.ac.cn) & dengxianjin_mtrc@caep.cn

**This PDF file includes:**

Materials and Methods

Figures S1 to S5

References

**Materials and Methods**

**Terahertz (THz) Multiplier Chain and Mixer**

The THz multiplier chain comprises multiple stages of frequency conversion and amplification to achieve high output power in the 410-510 GHz range. The system architecture includes two frequency doublers, two power amplifiers, a D-band frequency doubler, and a frequency tripler. The first two frequency doublers consist of an active doubler (HMC579) and a passive doubler (HMC1105), both based on Gallium Arsenide (GaAs) technology. Signal amplification is achieved using a GaAs monolithic microwave integrated circuit (CHA2080-98F) with a small-signal gain of approximately 22 dB, and a bare-die power amplifier (MAAP-011106) providing an additional gain of about 20 dB. These amplification stages collectively boost the signal to an output power of approximately 150 mW.

The D-band frequency doubler employs a planar Schottky diode (5VA30-13) fabricated on a thinned GaAs substrate. The design incorporates air-bridge interconnected mesas to minimize parasitic capacitance and features three series-connected anodes optimized for varactor-mode operation, thereby improving frequency multiplication efficiency. The subsequent frequency tripler integrates a capacitive Schottky diode (AP1-G1-0P95) with a junction capacitance of 13 fF and adopts a differential-balanced four-port configuration to suppress even-order harmonics and enhance spectral purity. With an input drive power of 50-80 mW, the tripler delivers an output power of approximately 1 mW, which is sufficient to drive the following THz mixer.

The THz mixer incorporates a resistive planar Schottky diode fabricated on a quartz substrate, featuring submicron-scale anode contacts to reduce junction capacitance and series resistance. In additional, the mixer integrates a compact, low-loss frequency-selective passive network composed of multi-section filtering sections. This design effectively suppresses undesired harmonics and enhances overall frequency conversion efficiency.

**Superconducting Mixer Based on SIS Junction**

Superconductor-insulator-superconductor (SIS) mixers operate based on the quantum tunneling of quasiparticles across an ultrathin insulating barrier between two superconducting electrodes. Due to the strong nonlinearity in their current-voltage (I-V) characteristics and the quantum nature of the tunneling process, SIS mixers offer ultralow noise temperatures and high conversion efficiency, making them the most sensitive heterodyne detectors in the 0.1-1 THz frequency range [1-3].

The SIS mixer developed in this work is based on niobium (Nb) parallel-connected twin-junction (PCTJ) SIS junctions. This configuration effectively reduces the geometric capacitance of the junction barrier, thereby improving impedance matching and enhancing mixer performance. To improve the edge quality of the SIS junction and reduce leakage currents, the photoresist layer on top of the junction is shrunk via oxygen ion treatment prior to SiO₂ deposition by RF magnetron sputtering. This new fabrication approach provides significantly improved performance compared to the conventional anodic oxidation method.

As shown in Fig. S2a, the mixer receives the local oscillator (LO) signal via a diagonal horn and the radio frequency (RF) signal through a smooth-walled horn. A full-height waveguide with dimensions of 255 μm (height) and 510 μm (width) is employed. The RF and LO signals are coupled into the SIS chip via bow-tie waveguide probes (Fig. S2b) and delivered to the PCTJs through quarter-wavelength superconducting microstrip lines (Fig. S2c). Each junction in the PCTJ structure is composed of a Nb/AlO/Nb trilayer with a diameter of 1.5 μm and a critical current density of 8 kA/cm². An integrated Nb/SiO₂/Nb tuning circuit is used to optimize impedance matching and maximize coupling efficiency.

The mixer chip is housed in a precision-machined waveguide block and cooled to 6 K using a compact, low-power-consumption pulse-tube cryocooler originally developed for space-based applications.

**Link Budget Estimation for Communication Experiment**

In the communication experiment, the transmitter delivers a total output power of only 15 μW (-18 dBm) at 481.4 GHz, radiated through a diagonal horn antenna with a gain of 25 dBi, yielding an effective isotropic radiated power (EIRP) of 7 dBm. On the receiver side, the received power at the 60-cm Cassegrain antenna is estimated to be -82.9 dBm. This estimate accounts for various loss mechanisms, including 4.8 dB of atmospheric attenuation, 147.7 dB of free-space path loss (FSPL), a 3 dB misalignment loss due to imperfect coupling between the horn and the Cassegrain optics, and a receiver antenna gain of 65.6 dBi. The FSPL is computed using Friis formula FSPL=32.45+20log_10_​(d)+20log​_10_(f), where d is the link distance in km and f is the carrier frequency in MHz [4]. The antenna gain is given by G=10log_10_(η(πD/​λ)^2^), where D is the antenna diameter, λ is the wavelength, and η is the antenna efficiency (assumed to be 0.4).

The achievable video bitrate is derived from the receiver sensitivity, which enables detection of signals as weak as -85.7 dBm, corresponding to a link margin of 2.8 dB. The receiver sensitivity is calculated using the expression P_min_=10log_10_(k_B_T_r_R_b_)+SNR_d_+L_d_, where T_r_=390 K is the receiver noise temperature, SNR_d_=10 dB is the required post-demodulation signal-to-noise ratio, and L_d_=2 dB accounts for the demodulation loss. Note that this expression is a combination of Shannon’s capacity limit [5] and classical link budget analysis based on the Friis transmission model [6]. Solving this equation yields an air-interface rate R_b_ of approximately 32 Mbps.

Moreover, we also conducted a theoretical link budget analysis for a proposed satellite-to-ground communication scenario. Specifically, we consider a system in which a spaceborne transmitter equipped with a 1-m antenna communicates with the ground-based 15-m XSMT telescope at 460 GHz. The gains of the transmitter and receiver antennas are estimated to be 69.7 dBi and 93.2 dBi, respectively. Given a transmitter output power of 10 dBm, the EIRP is calculated to be 79.7 dBm. Using Friis formula [6], the FSPL is estimated to be approximately 197.7 dB for a link distance of 400 km link at 460 GHz. Additional losses include 6.2 dB due to atmospheric attenuation (based on site-specific modeling at the XSMT location) and a 3 dB antenna pointing error. Consequently, the received power at the ground-based 15-m telescope is estimated as -34 dBm. Assuming a link margin of 3 dB, the required receiver sensitivity P_min_ is estimated to be -37 dBm. Using a receiver noise temperature of T_r_=200 K, a required post-demodulation signal-to-noise ratio of SNR_d_=10 dB, and a demodulation loss of L_d_=2 dB, and applying the same sensitivity expression, the corresponding air-interface rate R_b_ is estimated to be approximately 4500 Gbps.

Table I provides a comparison between the present THz communication experiment and the future satellite-to-ground link employing a 15-meter-diameter ground-based XSMT telescope operating at 460 GHz, paired with a 1-meter antenna onboard a satellite.

**Table I.** A comparison between this THz communication experiment and future satellite-to-ground communication based on the ground-based XSMT telescope (15 meters in diameter and operating at 460 GHz) and a space-based transmitter with a 1-m antenna.

| Parameter | Current Experiment | Future Scenario | Units |
| --- | --- | --- | --- |
| Frequency | 480.0 | 460.0 | GHz |
| Transmit Power | -18.0 | 10.0 | dBm |
| Transmit Antenna Gain | 25.0 | 69.7 | dBi |
| EIRP | 7.0 | 79.7 | dBm |
| Transmission Distance | 1.2 | 400.0 | km |
| Atmospheric Attenuation Factor | 3.8 | 0.016 | dB/km |
| Atmospheric Loss | 4.8 | 6.2 | dB |
| System Noise Temperature | 390.0 | 200.0 | K |
| Noise Figure | 3.7 | 2.28 | dB |
| Antenna Misalignment Loss | 3.0 | 3.0 | dB |
| Free-Space Path Loss | 147.7 | 197.7 | dB |
| Receiver Antenna Gain | 65.6 | 93.2 | dBi |
| Received Power | -82.9 | -34.0 | dBm |
| Demodulated SNR | 10.0 | 10.0 | dB |
| Link Margin | 2.8 | 3.0 | dB |
| Data Rate | 0.032 | 4500 | Gbps |

**Supplementary Figures**

**
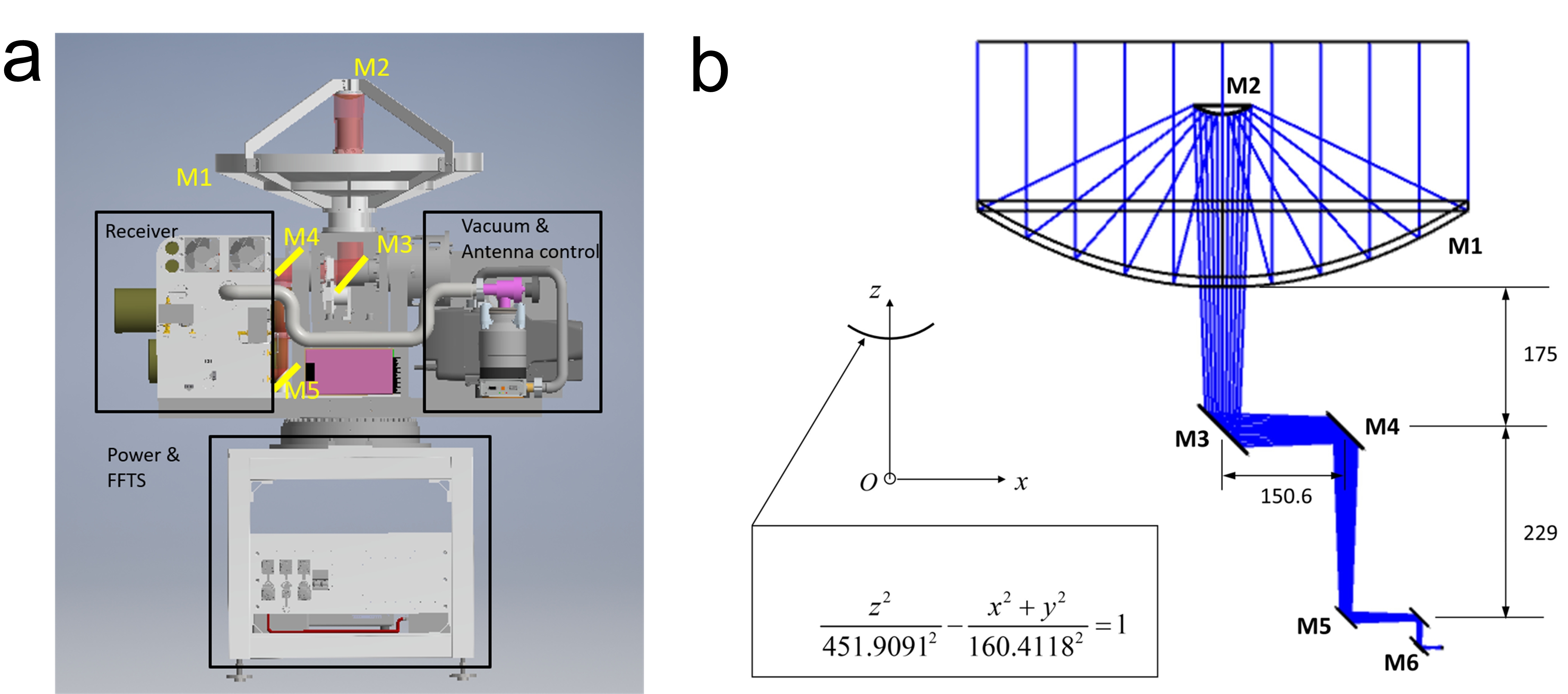
**

**Supplementary Figure S1.** The 60-cm portable telescope. (a) Schematic diagram of the telescope system, which includes a 60-cm Cassegrain antenna, a heterodyne receiver incorporating a Nb SIS mixer operating over the 430-530 GHz frequency range, a fast Fourier transform spectrometer (FFTS) with a spectral resolution of approximately 76 kHz, and integrated vacuum, control, and power supply modules. The SIS mixer is cooled by a compact, low-power-consumption pulse-tube (PT) cryocooler, providing a bath temperature of approximately 6 K. The PT cryocooler, initially developed for space applications, operates with an electric power consumption of only 400 W. (b) Signal pathway within the telescope. Incoming THz signal is collected by the 60-cm Cassegrain antenna, composed of a primary mirror (M1) and a secondary mirror (M2), and is then guided via a cascade of four flat mirrors (M3-M6) to the SIS mixer.

**
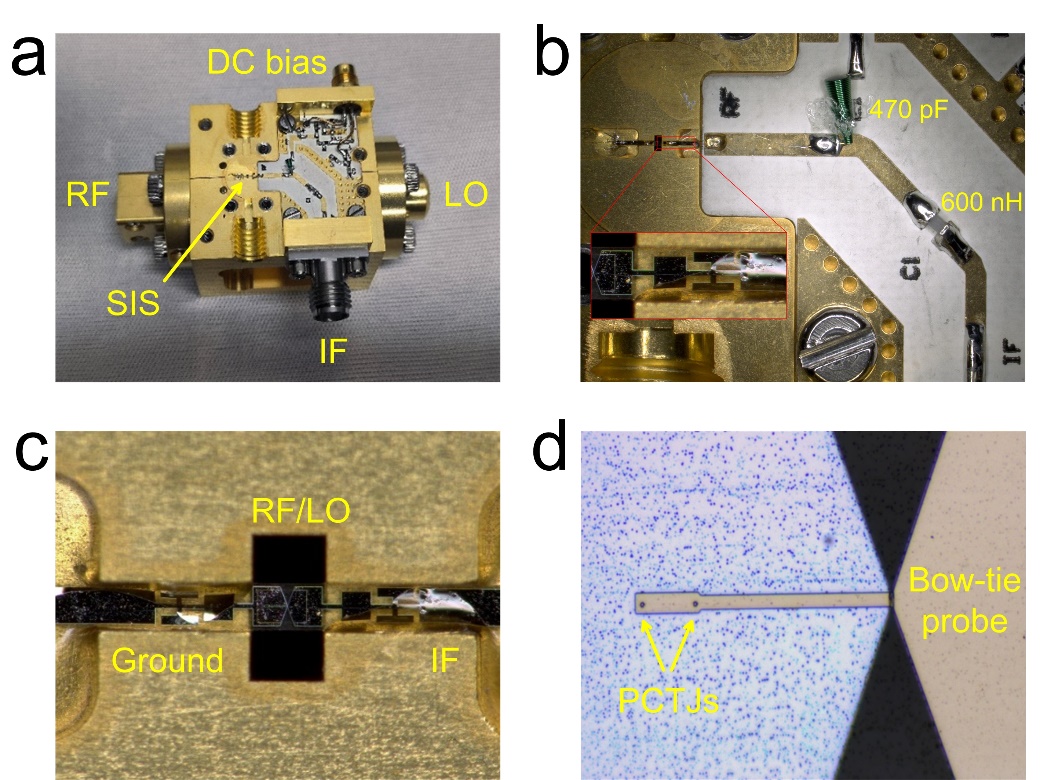
**

**Supplementary Figure S2.** Superconducting mixer based on SIS junction. (a) Photograph of the 430-530 GHz SIS mixer. The mixer integrates a diagonal horn for LO signal coupling and a smooth-walled horn for RF signal coupling. LO injection is realized via a waveguide directional coupler with a coupling factor of approximately 0.03 [7]. The SIS mixer is DC-biased using a four-wire configuration and operates without a mechanical tuner. A permanent magnet is employed to suppress the Josephson effect. (b) Photograph of the intermediate frequency (IF) circuit of the SIS mixer. A compact hammer-shaped choke filter is used in place of conventional high- and low-impedance choke filters to block RF and LO signals at the IF output, ensuring a clean IF signal. (c) Photograph of the SIS mixer chip within the mixer block. The RF and LO signals are delivered through a full-height waveguide with internal dimensions of 255 μm in height and 510 μm in width. The left end of the chip is grounded, while the right end connects to the IF output. (c) Close-up view of the SIS mixer chip. The chip incorporates Nb-based PCTJ SIS junctions. The RF and LO signals are coupled via a bow-tie waveguide probe and transmitted to the PCTJs through a quarter-wavelength superconducting microstrip line. Each PCTJ consists of a Nb/AlO/Nb tunnel junction with a diameter of 1.5 μm and a critical current density of 8 kA/cm^2^.

**
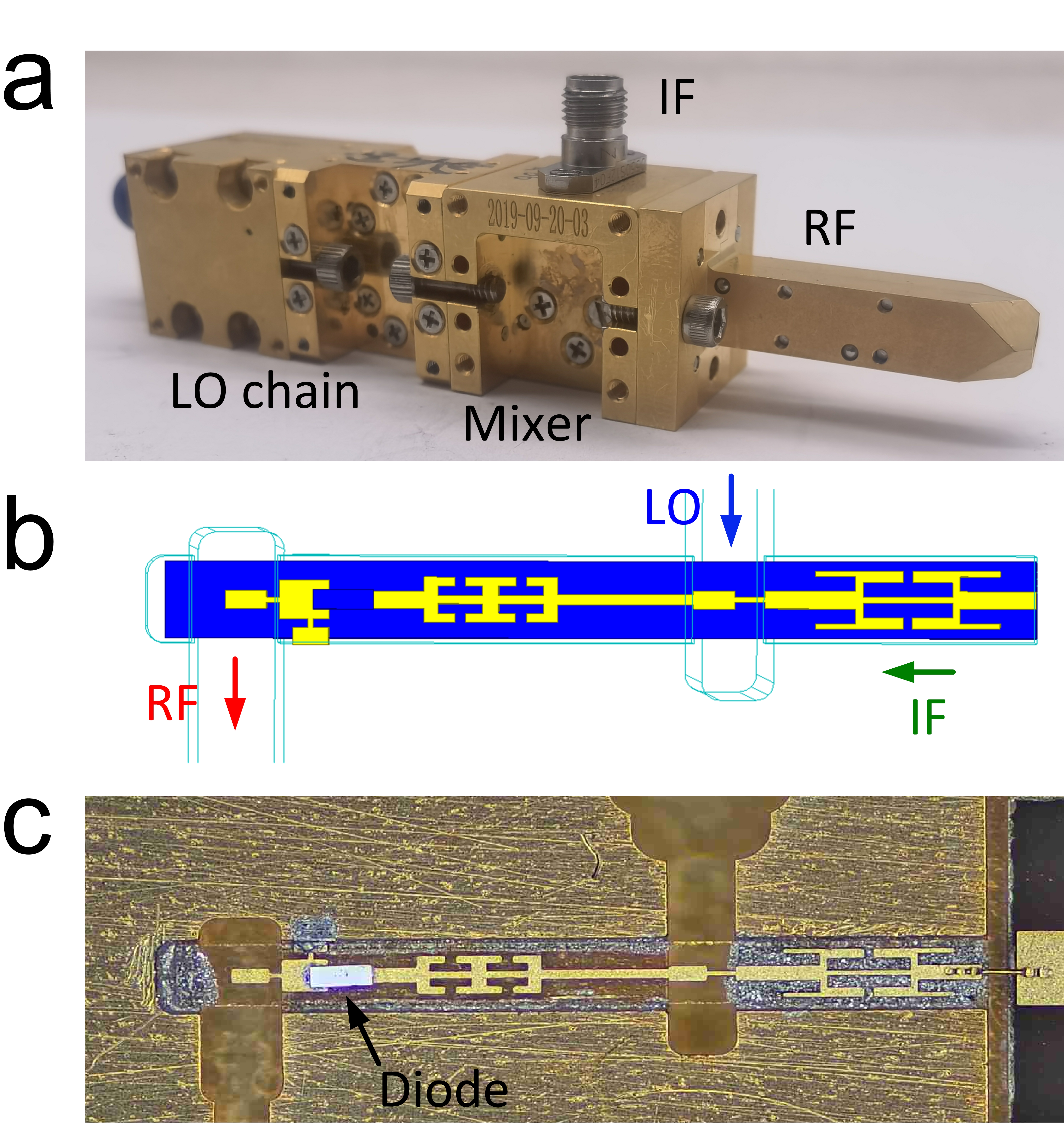
**

**Supplementary Figure S3.** The fully-electronic transmitter. (a) Photograph of the transmitter, which consists of a LO chain and a THz Schottky mixer. The LO chain integrates frequency multipliers and power amplifiers, delivering up to 1 mW of output power at 487.2 GHz. The THz Schottky mixer utilizes a planar resistive Schottky diode to upconvert a modulated signal at 5.8 GHz to a carrier frequency of 481.4 GHz. This compact, fully-electronic transmitter is particularly well suited for integration into satellite and airborne platforms. (b) High-Frequency Structure Simulator (HFSS) model of the THz Schottky mixer. The design features a low-loss, frequency-selective passive network with a multi-section filtering topology, which effectively suppresses undesired harmonics and enhances frequency conversion efficiency. (c) Internal structure of the THz Schottky mixer. A planar resistive Schottky diode is integrated on a quartz substrate with submicron-scale anode contacts, enabling a typical conversion loss of approximately 13 dB.

**
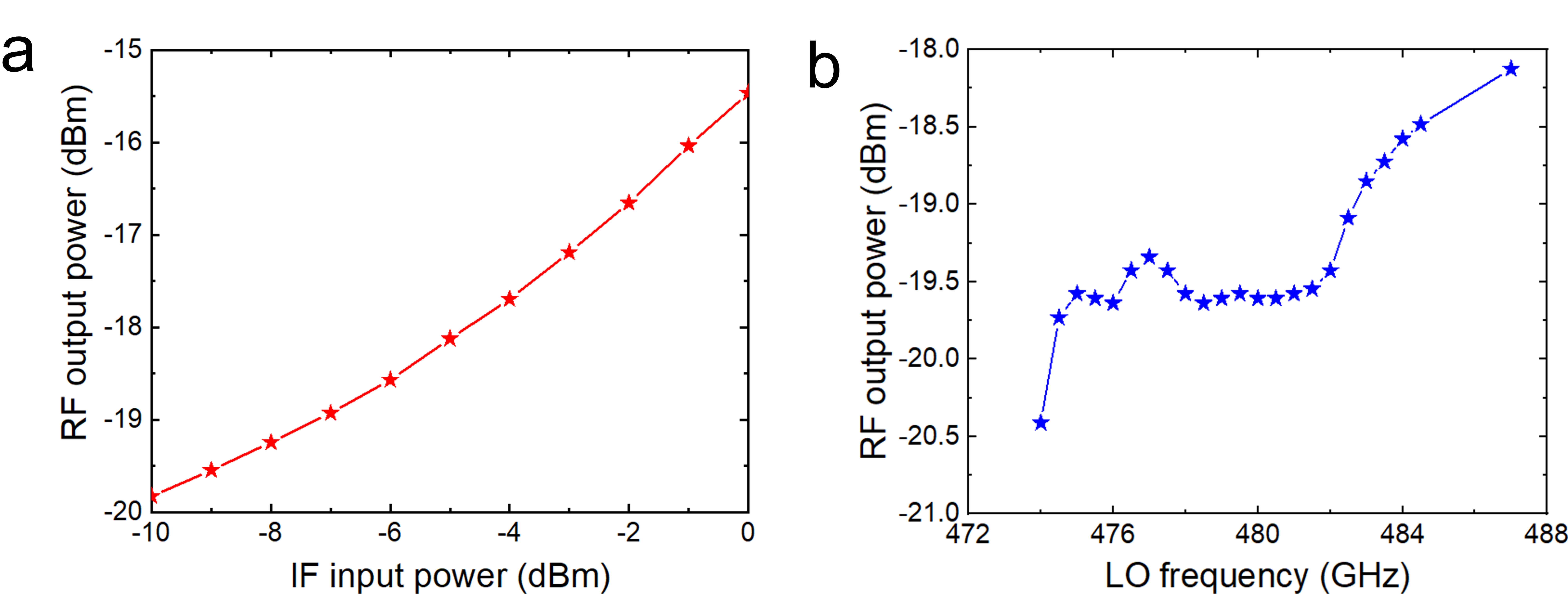
**

**Supplementary Figure S4.** Measured RF output power. (a) Measured RF output power of the THz Schottky mixer as a function of IF input power. The measurements were conducted with the LO frequency fixed at 487.2 GHz and the IF frequency set to 5.8 GHz. As the IF input power increases from -10 dBm to 0 dBm, the RF output power varies by about 4.5 dB, exhibiting an approximately linear response, indicative of the mixer’s stable upconversion behavior within this power range. (b) Measured RF output power of the THz Schottky mixer as a function of the LO frequency, with the IF input power held constant at -5 dBm. The RF output power displays very weak dependence on the LO frequency, implying the mixer’s capability for broadband and frequency-stable operation.

**
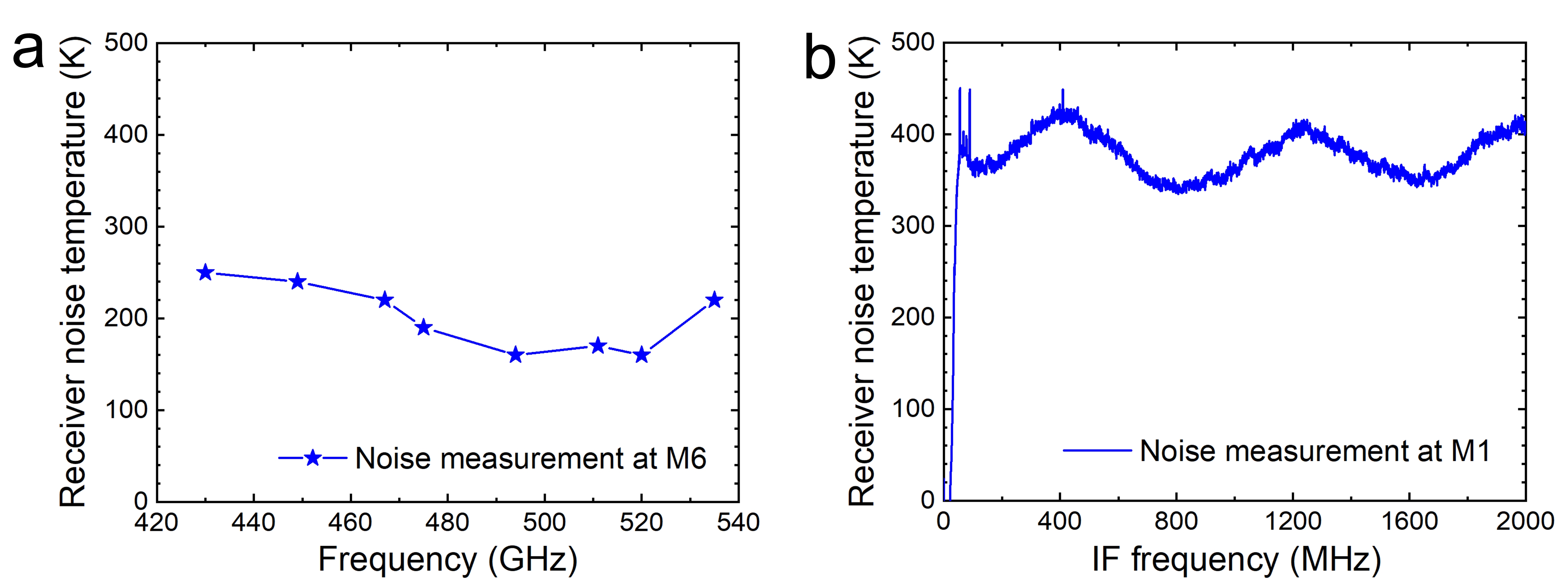
**

**Supplementary Figure S5.** Double sideband (DSB) noise temperature of the SIS receiver. (a) Measured noise temperature of the SIS receiver at the Dewar window, located after the flat mirror M6, across the 430-530 GHz frequency range. The measurements were performed using the standard Y-factor method, with 300 K and 77 K blackbody loads serving as calibration references. The Y-factor is defined as the ratio of the IF output powers under the two thermal load conditions, Y=P_hot_/P_cold_, where P_hot_ and P_cold_ denote the IF output powers corresponding to the 300 K and 77 K loads, respectively. The receiver noise temperature is calculated by T_rec_=(T_hot_-T_cold_*Y)/(Y-1). The reported noise temperature represents an average over the IF band from 0.2 to 2.0 GHz. An optimal DSB noise temperature of 200 K was obtained, demonstrating the excellent sensitivity of the SIS mixer. Major noise contributions stem from the Dewar window and infrared filter, the SIS mixer itself, and the subsequent IF amplification chain. After accounting for ~2 dB of RF loss from the Dewar window and infrared filter and ~8 K of noise contribution from the cryogenic low-noise amplifier (LNA), the intrinsic noise temperature of the SIS mixer was deduced to be approximately 100 K. (b) Measured noise temperature of the entire telescope as a function of IF frequency, with the LO frequency fixed at 490 GHz. The measurements were taken at the position between the primary mirror M1 and the secondary mirror M2 of the Cassegrain antenna. The noise temperature is about 390 K, higher than the value recorded at the Dewar window. This increase is primarily due to optical losses from the reflective mirrors (M2-M6) and atmospheric water vapor absorption along the optical path. The entire system is cooled using a low-power-consumption cryocooler, which maintains a bath temperature of about 6 K, higher than the 4.2 K typically used for Nb SIS mixers. This leaves clear room for further improvement in noise performance, for instance by adopting high-energy-gap SIS mixers [8] or employing a conventional 4 K cryocooler as used in large-aperture THz observatories. Furthermore, periodic oscillations in the measured noise temperature, with a characteristic period of roughly 800 MHz, are likely attributed to impedance mismatches between the SIS mixer and the cryogenic LNA.

**References**

[1] Liu BL, Liu D, Yao M *et al.* Terahertz high-sensitivity SIS mixer based on Nb-AlN-NbN hybrid superconducting tunnel junctions. *Chin Phys B* 2024; **33**: 058501.

[2] Gao X, Du J, An JP*.* Cryogenic receiver frontends for high sensitivity for terahertz communication applications. *15th Global Symposium on Millimeter-Waves & Terahertz (GSMM)* 2024; 174-176.

[3] [Kohjiro](javascript:;) S, [Kikuchi](javascript:;) K, [Maezawa](javascript:;) M *et al.* A 0.2–0.5 THz single-band heterodyne receiver based on a photonic local oscillator and a superconductor-insulator-superconductor mixer. *Appl Phys Lett* 2008; **93**: 093508.

[4] Shah S, Ashraf M. Signal path loss measurement for future terahertz wireless propagation links. *Int J Eng* 2018; **5**: 193-197.

[5] Shannon CE. Communication theory of secrecy systems. *Bell System Technical Journal* 1949; **28**: 656-715.

[6] Friis HT. A note on a simple transmission formula. *Proceedings of the IRE* 1946; 34: 254-256.

[7] Khudchenko A, Hesper R, Baryshev AM *et al.* Design and performance of a sideband separating SIS mixer for 800-950 GHz. *IEEE Trans Terahertz Sci Technol* 2019; 9: 532-539.

[8] Li J, Takeda M, Wang Z *et al.* Low-noise 0.5 THz all-NbN superconductor insulator superconductor mixer for submillimeter wave astronomy. *Appl Phys Lett* 2008; **92**: 222504.
